# Orally-Induced Intestinal CD4^+^ CD25^+^ FoxP3^+^ Treg Controlled Undesired Responses towards Oral Antigens and Effectively Dampened Food Allergic Reactions

**DOI:** 10.1371/journal.pone.0141116

**Published:** 2015-10-30

**Authors:** Paola Lorena Smaldini, María Lucía Orsini Delgado, Carlos Alberto Fossati, Guillermo Horacio Docena

**Affiliations:** Instituto de Estudios Inmunológicos y Fisiopatológicos-IIFP, Facultad de Ciencias Exactas, Universidad Nacional de La Plata y Consejo Nacional de Investigaciones Científicas y Técnicas, La Plata, Argentina; Wayne State University, UNITED STATES

## Abstract

The induction of peripheral tolerance may constitute a disease-modifying treatment for allergic patients. We studied how oral immunotherapy (OIT) with milk proteins controlled allergy in sensitized mice (cholera toxin plus milk proteins) upon exposure to the allergen. Symptoms were alleviated, skin test was negativized, serum specific IgE and IgG1 were abrogated, a substantial reduction in the secretion of IL-5 and IL-13 by antigen-stimulated spleen cells was observed, while IL-13 gene expression in jejunum was down-regulated, and IL-10 and TGF-β were increased. In addition, we observed an induction of CD4^+^CD25^+^FoxP3^+^ cells and IL-10- and TGF-β-producing regulatory T cells in the lamina propria. Finally, transfer experiments confirmed the central role of these cells in tolerance induction. We demonstrated that the oral administration of milk proteins pre- or post-sensitization controlled the Th2-immune response through the elicitation of mucosal IL-10- and TGF-β-producing Tregs that inhibited hypersensitivity symptoms and the allergic response.

## Introduction

The prevalence of food allergies has increased over the last decade and constitutes a highly morbid disorder. [1]. The restriction diet represents the current treatment for milk-allergic children, but it may be difficult to comply for multiple reasons: misunderstanding or incomplete information in food labeling, nutritional inadequacy of dairy substitutes, growth retardation, eating disorders and psychosocial problems [2]. For this reason, efforts have been made to develop alternative therapies that complement the avoidance strategy and restore an adequate immune management of food antigens. As there are no currently approved and standardized therapies for food allergies, patients are instructed to strictly avoid the allergenic food and ensure a ready access to epinephrine and anti-histamine [3].

Evidence of a lack of oral tolerance in food allergic patients [4,5] has increased the interest in oral immunotherapy (OIT) as an option for a disease-modifying therapy. Although it is an experimental treatment, several clinical trials have shown promising results. However, safety and efficacy are not yet shown and further research is needed to identify the network of regulatory pathways that are induced to limit the tissue inflammation [6,7]. Although OIT has shown to be effective in inducing clinical desensitization to some food allergens, the mechanisms underlying these therapeutic procedures have not been completely described [8]. OIT to cow´s milk allergy has been actively investigated [9–11] and, despite periods of withdrawals, it was shown that no symptoms were provoked following milk ingestion [12,13]. It is known that food-specific regulatory T cells are generated in the gastrointestinal tract, although the exact mechanism of action has not been unveiled [14].

Dissecting the mechanisms underlying this phenomenon in food allergies is difficult in humans, and animal model studies provide new clues for understanding and controlling the immune response in the compromised mucosa. In this work, we used an IgE-mediated mouse model of food allergy to study the local and systemic regulatory mechanisms of protection promoted by OIT. The repeated oral administration of cow’s milk proteins (CMP) induced lamina propria regulatory T cells that controlled the allergic reaction towards oral antigens through the production of IL-10 and TGF-β. The adoptive transfer of CD4^+^CD25^+^FoxP3^+^ Treg confined the protection mechanism and the depletion of CD25^+^ T cells resulted in pronounced disease exacerbation, thus confirming that Treg have an essential role in resolving food allergy in our model.

## Materials and Methods

### Ethics statement

All experimental protocols of this study were conducted in strict agreement with international ethical standards for animal experimentation (Helsinki Declaration and its amendments, Amsterdam Protocol of welfare and animal protection and National Institutes of Health, USA NIH, guidelines: Guide for the Care and Use of Laboratory Animals). Anesthetized mice (isoflurane 5%) were killed by cervical dislocation by experienced research personnel, which performed it humanely and affectively. All efforts were made to alleviate suffering during the whole experiment. The protocols of this study were approved by the Institutional Committee for the Care and Use of Laboratory Animals from University of La Plata (Protocol Number: 017-00-15).

### Animals, sensitization and challenge

**Fig 1:**
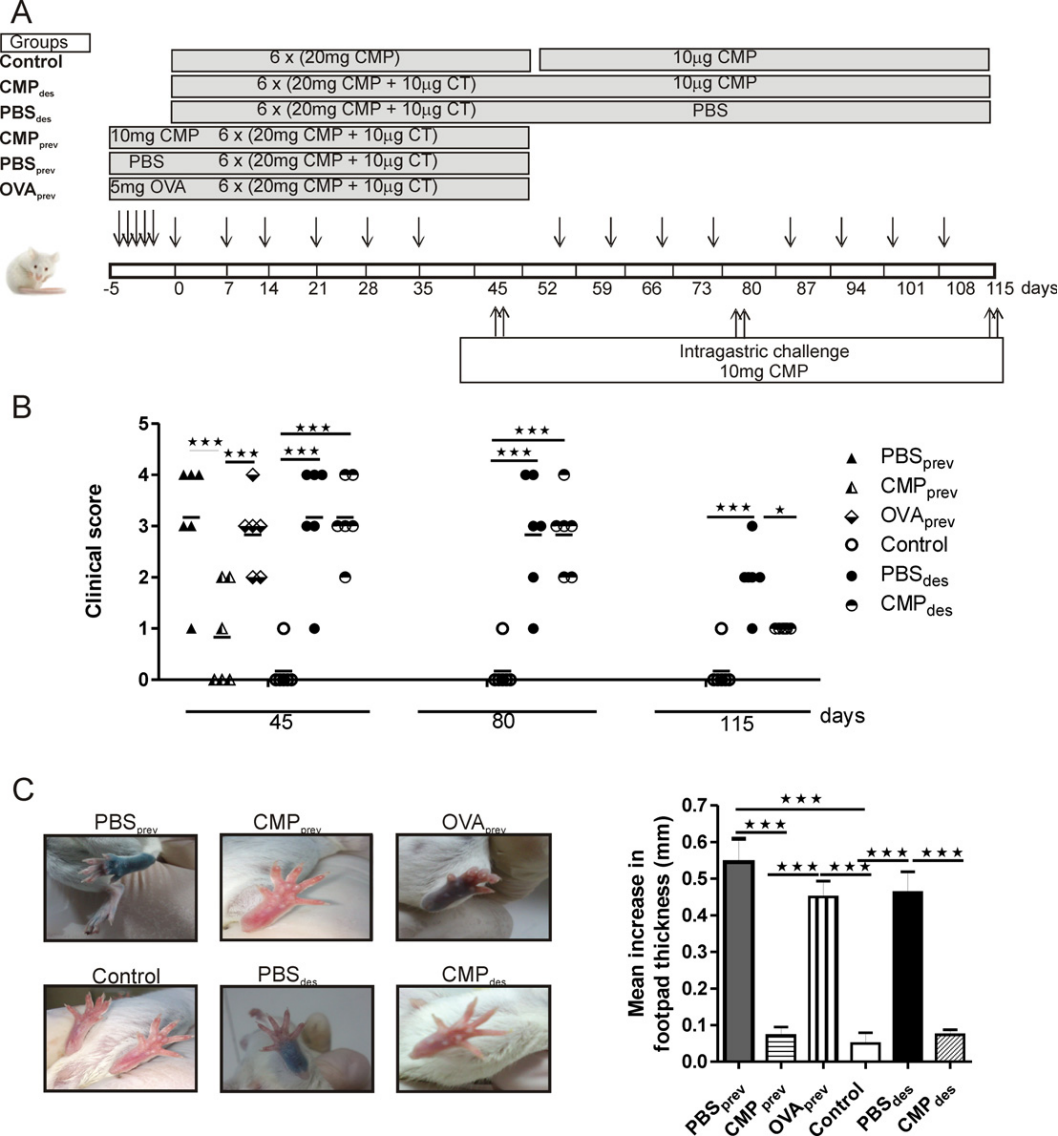
Experimental design and i*n vivo* responses to the oral administration of CMP in the food allergy mouse model. **A**, Schematic drawing of the experimental protocol in the BALB/c mice (*n* = 6/group). **B**, Clinical scores corresponding to symptoms elicited following the oral challenges with milk proteins. **C,** Skin tests in control, sensitized and treated mice. Swelling of footpad was quantified and data are expressed as the mean values ±SEM. The results correspond to a single experiment, which is representative of three separate experiments that had similar results. One-way ANOVA was used because all the data had normal distribution and equal variances ***p<0.005, **P<0.01. *CMP*, cow’s milk proteins; *PBS*_*prev*_, control group that received PBS prior sensitization; *CMP*_*prev*_, preventive treatment with CMP; *PBS*_*des*_, control group that received PBS after sensitization; *CMP*_*des*_, desensitization with CMP; *OVA*_*prev*_, preventive treatment with ovalbumin.

Male 6- to 8-week old BALB/c mice were sensitized according to Smaldini et al. [15]. Briefly, mice received 6 weekly intragastric (ig) doses of 20 mg of skimmed CMP and 10 μg of cholera toxin (CT) (Sigma Aldrich, St. Louis, USA) in bicarbonate buffer per mouse (n = 6/group). Ten days after the final boost, mice were ig challenged with 10 mg of CMP on two consecutive days. Twenty-four hours later animals were sacrificed by cervical dislocation. Control group of mice received only 20 mg CMP (without CT) during the sensitization phase and 10 mg during the treatment phase. The experimental design is shown in Fig 1A.

### Oral administration of CMP as treatment

Mice were divided into two groups for treatment: those who received 10 mg/mouse/day of CMP by gavage during five days prior to sensitization (CMP_prev_), and those who were sensitized and then treated with CMP_ig_ (10 μg/mouse/per dose) once per week for 8 weeks (CMP_des_). Control groups of mice consisted of mice receiving PBS_ig_ instead of CMP (PBS_prev_ and PBS_des_) (Fig 1A). In addition, as a control of specificity other mice preventively received OVA (5 mg/per dose) and then were sensitized with CMP plus CT as described (OVA_prev_). Animals were grouped in 6 mice per condition and experiments were repeated at least twice.

### *In vivo* evaluation of the allergic reaction

#### Clinical symptoms

Symptoms were observed 45 minutes after challenges with CMP_ig_ in a blinded fashion by 2 independent investigators (Table 1).

**Table 1:** Clinical scores assigned to triggered symptoms following the oral challenges.

Score	Symptoms
**0**	**No symptoms**
**1**	**Scratching and rubbing around the snout and head**
**2**	**Puffiness around the eyes and mouth, pilo-erection, reduced activityand/or decreased activity with increased respiratory rate**
**3**	**Hyperreactivity followed by respiratory distress, cyanosis around the snout and tail**
**4**	**No activity upon stimuli, convulsion**
**5**	**Death**

#### Cutaneous tests

Mice were injected with 20 μg of CMP in 20 μl of sterile saline in one footpad, and saline, in the contra-lateral footpad as a negative control. Then, mice were injected intravenously (*iv*) with 100 μl of 0.1% Evans blue dye (Anedra, Buenos Aires, Argentina). The presence of blue color 10–20 minutes after the injection was considered a positive test and footpad swelling was measured with a digital micrometer.

### *In vitro* evaluation of the allergic reaction

#### Serum specific antibodies

CMP-specific IgE was measured by EAST, and IgG1 and IgG2a were measured by ELISA as previously described [15–18]. All samples were run in the same experiment.

#### Cytokine response of spleen cells to antigen stimulation

4x10^6^ spleen cells/ml were plated in complete medium (RPMI-1640 supplemented with 10% FBS, 100 U/ml penicillin and 100 μg/ml streptomycin) with CMP (350 μg/ml) for 72 hours. Cytokines (IL-5, IL-13, IFN-γ) were measured in supernatants by ELISA (Invitrogen, Carlsbad, CA, USA) (17).

#### Cytokine quantification by ELISA in the jejunum

Frozen sections of jejunum were minced and cells were treated with lysis buffer (10 mM Tris-HCl, 150 mM NaCl, 1% NP-40, 10% Glycerol, 5 mM EDTA and a protease inhibitor cocktail-Sigma). Homogenate was sonicated and the supernatant was collected. IL-10 and TGF-β were determined by ELISA (eBioscience, San Diego, CA, USA).

#### Real time RT-PCR for gene expression

The mRNA expression was determined by real-time quantitative PCR on an *ABI prism* sequence detection system using *SYBRGreen* fluorescence (BioRad, Hercules, CA, USA). β-actin was used as a housekeeping gene and the fold change in the mRNA expression was defined as the ratio of the normalized values in sensitized mice to that in control mice, as previously described [19]. The genes of interest were: *IFN-γ*, *IL-5*, *IL-13*, *IL-10*, *TGF-β* and *FoxP3*.

#### Cell isolation from gut

Cells were isolated from lamina propria (LP) as described in [20]. Briefly, the entire gut was removed from mice and the second half of the small bowel was excised (jejunum). The epithelial compartment was removed (incubation with HBSS and EDTA 1mM) and then tissue was treated in complete RPMI-1640 medium with 1mg/ml of collagenase and 10U/ml of DNAse. Cell debris were removed by filtration and the cell suspension was used to characterize lamina propria cells. Cells from mesenteric lymph nodes (MLN) were obtained by digestion with collagenase in RPMI for 30 minutes at 37°C. Cell suspensions were filtered and washed in RPMI.

*Flow Cytometry for Cell Characterization*: Cells from MLN and LP were stimulated with recombinant mouse IL-2 (20 ng/ml, Preprotech, NJ, USA) for 12 hs at 37°C with 3 μg/ml of Brefeldin A (eBioscience) added for the last 4 hs to prevent egress of newly synthesized proteins from endoplasmic reticulum. Cells were washed and stained for anti-CD4 (PerCyP 5.5) and anti-CD25 (PE or FITC) (eBioscience). Cells were washed and pre-incubated with fixation/permeabilization solution (eBioscience) 20 minutes at 4°C. For intracellular staining cells were treated with Staining Intracellular kit (eBioscience) and anti-FoxP3 (APC or PE), anti-IL-10 (APC) or anti-TGF-β (APC) (eBioscience). Fluorescence acquisition was performed with a FACScalibur cytometer using QuestProCell software. The gating strategy for cell analysis consisted on a lymphocyte gate based on SSC-H vs FSC-H parameters, followed by SSC-H vs CD4 fluorescence or followed by CD25 vs CD4 fluorescence. The CD4^+^ lymphocyte population was gated as CD25 vs FoxP3, and CD4^+^ CD25^+^ lymphocyte population was gated as FoxP3 vs IL-10 or TGF-β expression. The data were analyzed with the FlowJo software.

### Adoptive cell transfer for acquired tolerance in sensitized mice

Peripheral inducible Treg were generated *in vitro* or *in vivo* and then transferred to naïve animals, which were then sensitized as described.

#### *In vitro* generation of TGF-β-induced regulatory T cells (Tregs)

Spleen was removed from naïve mice, cell suspension was prepared and CD4^+^CD25^-^ cells were aseptically sorted (BD FACS Aria II) using anti-CD4 and anti-CD25 antibodies (eBioscience). Sorted cells were stimulated with anti-CD3/anti-CD28 (2.5 μg/ml and 2 μg/ml respectively; eBioscience) in the presence of IL-2 (20 ng/ml, Preprotech) and TGF-β (5 ng/ml, Preprotech) for 5 days. The phenotype of *ex vivo* generated Treg cells was analyzed by flow cytometry (FoxP3 and CD25 expression) and ELISA (IL-10 concentration in the supernatant of differentiated cells). For induction of tolerance 1x10^6^ differentiated cells were *iv* injected into recipient mice, which were then sensitized as described (n = 5/group).

#### *In vivo* induction of regulatory T cells

Donor mice were daily administrated CMP (10 mg/mouse) or saline during 5 days, and then sensitized with CMP plus CT as described. Lymphocytes isolated from MLN were *iv* injected (4x10^6^ cells) into recipient mice, which were then sensitized as described. Other recipient mice received MLN cells from sensitized donor mice treated with PBS_ig_ as control.

To confirm whether CD4^+^ CD25^+^ Treg mediated regulatory mechanisms that reversed the allergic response in sensitized mice, CD25^+^ cells were depleted prior to adoptive cell transfer. Cells isolated from sensitized mice (PBS_prev_) as control (with low number of Treg) or tolerized mice (CMP_prev_) (with high number of Treg) were *ex vivo* incubated with rat anti-mouse CD25 (eBioscience) or rat IgG isotype (eBioscience) plus rat serum during 40 minutes at 4°C. The remaining cells were washed and the phenotype was analyzed by flow cytometry. Cells were injected into recipient naïve mice (n = 5/group) which were then sensitized as described. As additional control, recipient mice were injected with saline and then sensitized.

### Statistical analysis

All the statistical analyses were performed using GraphPad Prism 5 software. The significance of the difference was determined using an independent-sample t-test or ANOVA followed by Bonferroni`s post-test. A p-value <0.05 was regarded as statistically significant.

## Results

### The intragastric administration of CMP ameliorated the allergic reactions

We followed two immunomodulatory strategies: prevention (CMP_prev_) and desensitization (CMP_des_) (Fig 1 A). Animals were sensitized with milk proteins and hypersensitivity reactions observed immediately after the oral challenges with CMP were scored (Fig 1B). At day 45, sensitized mice exhibited clinical signs corresponding to allergic sensitization (PBS_prev_, PBS_des_ and CMP_des_), whereas control mice showed no symptoms. At this time point, CMP_prev_ mice were protected from allergy and CMP_des_ mice showed a high score (desensitization treatment started at day 52). After the first round of treatment (day 80), CMP_des_ mice still showed high scores; and following the second round of treatment (day 115), CMP_des_ mice presented a diminished average clinical score. In addition, mice pre-treated with OVA (OVA_prev_), and then sensitized with CMP plus CT, showed similar scores to sensitized mice.

In concordance with these findings, we showed the presence of IgE-sensitized mast cells in the footpads of mice. As shown in Fig 1C, skin tests were positive in sensitized (PBS_prev_ and PBS_des_) and OVA_prev_ mice, whereas CMP_prev_ and CMP_des_ mice showed negative results. Footpad swelling was statistically lower in CMP-treated mice compared with sensitized mice (0.54±0.04, 0.07±0.02, 0.46±0.05, and 0.07±0.01 PBS_prev_, CMP_prev_, PBS_des_ and CMP_des_, respectively) or OVA_prev_ mice (0.43±0.03).

### Systemic and mucosal Th2-biased specific immune responses were controlled with the intragastric administration of CMP in sensitized mice

**Fig 2:**
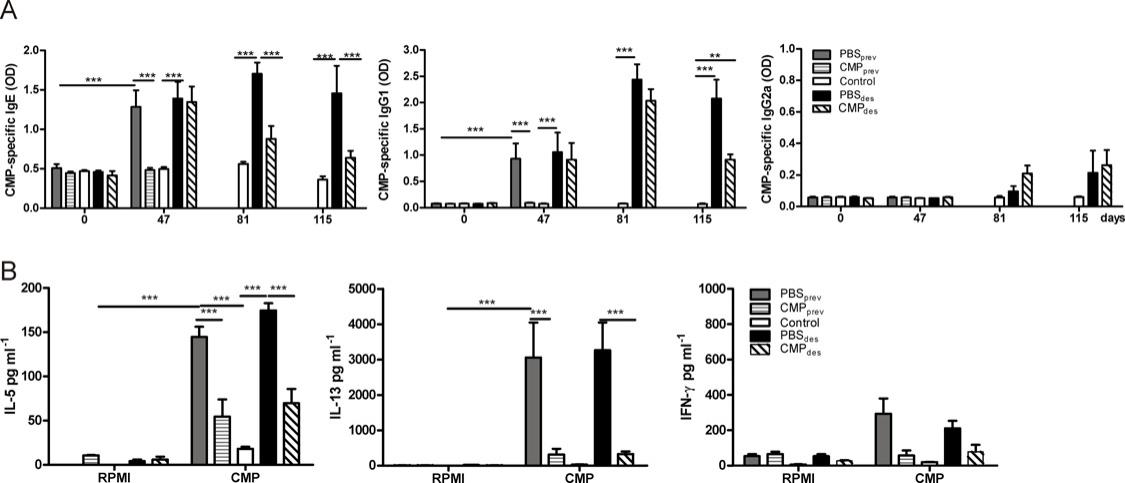
Serum specific isotypes and cytokines by ELISA. **A**, Serum CMP-specific IgE, IgG1 and IgG2a. **B**, Cytokines in the supernatants of spleen cells stimulated with CMP or medium for 72 hs. These results are representative of two independent experiments with similar results. The data are expressed as the mean values ±SEM. Two-way ANOVA was used because all the data had normal distribution and equal variances. ***P<0.001, **P<0.01.

Serum CMP-specific immunoglobulins and cytokines secreted by CMP-stimulated spleen cells were studied (Fig 2). CT-driven sensitization (PBS_prev_ and PBS_des_) induced sustained high milk-specific IgE and IgG1 antibodies with no induction of specific IgG2a, even 80 days after sensitization. Control mice and pre-treated animals showed no increase in any serum specific isotype. CMP_des_ mice showed a reversion of specific IgE secretion after the second round of treatment. Besides, treated animals showed no significant induction of serum CMP-specific IgG2a level. OVA_prev_ mice showed the same serum pattern as PBS_prev_ (data not shown).

Cytokine analysis showed that the levels of Th2 cytokines were significantly controlled in orally treated mice. As seen in Fig 2B, milk-stimulated spleen cells of sensitized mice (PBS_prev_ and PBS_des_) secreted elevated amounts of Th2 cytokines (IL-5 and IL-13), which were significantly suppressed in treated animals. Levels of IFN-γ were low in all animals.

**Fig 3:**
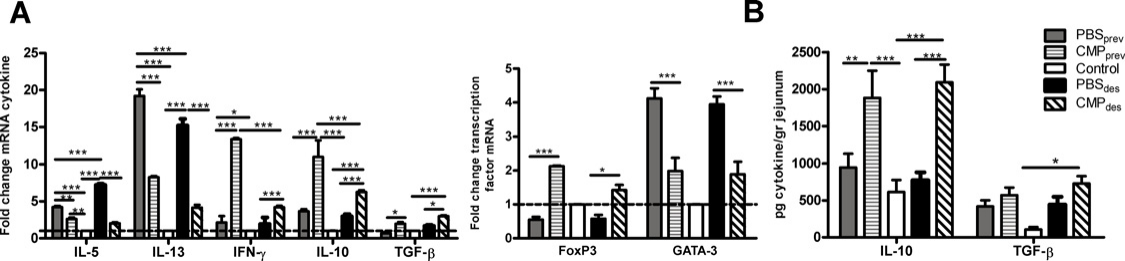
Cytokine expression and Treg analysis. **A**, mRNA expression of cytokines (IL-5, IL-13, IFN-γ, IL-10, TGF-β and FoxP3) in the jejunum by quantitative PCR. β-actin was used to standardized the total amount of cDNA and the fold change in mRNA expression was defined as the ratio of the normalized values corresponding to sensitized or treated mice to that of control mouse. **B,** TGF-β and IL-10 protein levels in the jejunum by ELISA. The data are expressed as the mean values ±SEM. These results are representative of two independent experiments with similar results. Two-way ANOVA was used because all the data had normal distribution and equal variances. ***P<0.001, **P<0.01, *P<0.05.

To address the effect of CMP_ig_ administration on the intestinal mucosa we assessed the gene expression corresponding to *IL-5*, *IL-13*, *IFN-γ*, *IL-10*, *TGF-β* and *Foxp3* in jejunum (Fig 3A). Sensitized mice (PBS_prev_ and PBS_des_) displayed increased expression of *IL-5* and *IL-13* compared with control mice (fold change of gene expression relative to control mice is depicted). Treatments with CMP_ig_ (CMP_prev_ and CMP_des_) significantly abrogated the CT-driven induction of *IL-5* and *IL-13*, with a significant induction of *IFN-γ*, *IL-10* and *TGF-β*. In addition, these mice showed increased levels of *FoxP3* (Fig 3B). When suppressor cytokines were analyzed at the protein level we found that IL-10 was increased in jejunum of CMP_prev_ and CMP_des_ mice. TGF-β was significantly augmented only in CMP_des_ mice (Fig 3B).

### The intragastric administration of CMP induced intestinal Tregs

**Fig 4:**
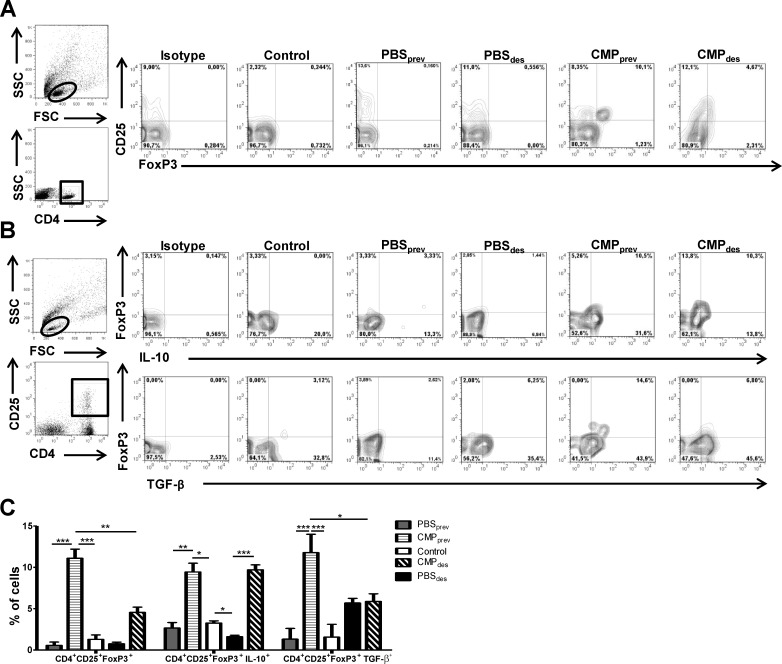
Regulatory T cells characterization by flow cytometry. **A**, Representative staining of CD25^+^ FoxP3^+^ cells gated on CD4^+^ lymphocytes (T cells) in the lamina propria by flow cytometry. B, Representative staining of IL-10^+^ FoxP3^+^ and TGF-β^+^ FoxP3^+^ cells gated on CD4^+^CD25^+^ lymphocytes (anti-CD4 PE-Cy5, and anti-CD25 FITC or PE for membrane staining, and anti-FoxP3 PE or APC, anti-IL-10 APC and TGF-β APC for intra-cytoplasmic staining, isotype controls were included). Frequencies of cells are expressed as the mean values ±SEM. These results are representative of two independent experiments with similar results. Two-way ANOVA was used because all the data had normal distribution and equal variances. ***P<0.001, **P<0.01, *P<0.05.

Based on previous findings we analyzed the presence of Tregs in lamina propria by flow cytometry. As shown in Fig 4A we found elevated percentages of CD4^+^CD25^+^FoxP3^+^ cells of treated mice (11.1±1.1% and 4.55±0.5% CMP_prev_ and CMP_des_ respectively) compared to control or sensitized mice (0.59±0.4, 0.17±0.2, and 0.55±0.3% control, PBS_prev_ and PBS_des_ respectively. Tregs as producers of regulatory cytokines were also analyzed (Fig 4B), and we found a significant high frequency of IL-10-producing CD4^+^CD25^+^FoxP3^+^ T cells in lamina propria of treated mice (9.44±0.73% CMP_prev_ and 9.70±0.41% CMP_des_), whereas diminished frequencies were observed in control and sensitized mice (1.62±0.03% PBS_prev_ and 2.68±0.11% PBS_des_). However TGF-β-producing CD4^+^CD25^+^FoxP3^+^ T cells were only found increased in CMP_prev_ mice (11.8±3.07%) compared with PBS_prev_ (1.32±0.91%) as shown in Fig 4C.

Concomitant with the increase in the frequency of Treg, we observed a modulation of CD4^+^CD25^+^ effector Tcells in mice that were pre-treated with CMP (13,07±2,03 vs 8,09±0,28%, PBS_prev_ vs CMP_prev_, p<0,05).

### Food allergy was suppressed by adoptive transfer of Tregs

#### *In vitro*-differentiated Tregs

**Fig 5:**
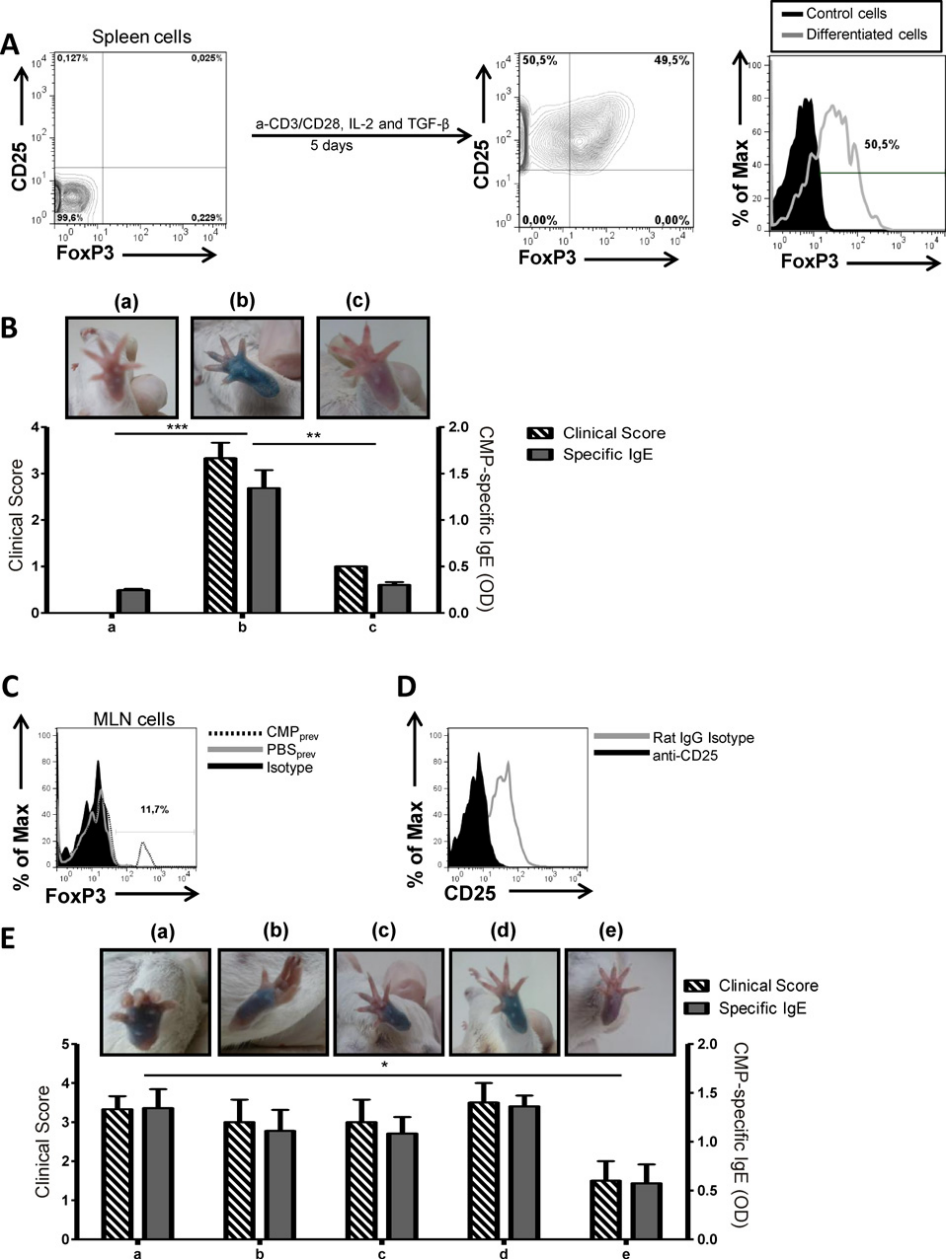
Adoptive transfer of regulatory T cells. Naïve mice that were transferred with different cells, were subsequently sensitized with CMP and CT, and finally challenged with CMP_ig_ (n = 5/group). **A**, Transfer of *in vitro*-differentiated Treg. Sorted naïve CD4^+^CD25^-^ spleen cells were stimulated with a-CD3/a-CD28, IL-2 and TGF-β for 5 days. CD25^+^FoxP3^+^ cells gated on CD4^+^ lymphocytes were quantified (histogram) by flow cytometry and transferred to naïve receptor mice. **B,** Skin test, symptoms scored following the oral challenge and serum CMP-specific IgE in receptor mice. a) Control mice: naïve mice only received PBS_iv_; b) Control mice: naïve mice received PBS_iv_ and were sensitized; c) naïve mice received differentiated-Treg and were sensitized. **C,** *In vivo*-induced Treg in mice that were orally given daily dose of CMP and then sensitized (CMP_prev_). As control, donor mice received daily dose of PBS_ig_ and then were sensitized (PBS_prev_). MLN cells were isolated from donor mice and FoxP3^+^ cells (gated from CD4^+^CD25^+^ lymphocytes) were evaluated and quantified by flow cytometry. **D**, CD25 depletion was performed on MLN cells and evaluated by flow cytometry. Depleted and not depleted cells were adoptively transferred to receptor mice, which were then sensitized. **E**, Skin test, symptoms scored following the oral challenge and serum CMP-specific IgE in receptor animals. a) Control mice: naïve recipient mice received PBS_iv_ and then were sensitized; b) Control mice: receptor mice that received MLN cells depleted with a-CD25 isolated from donor PBS_prev_ animals and then were sensitized; c) Receptor mice that received MLN cells depleted with a-CD25 isolated from donor CMP_prev_ animals and then were sensitized; d) Receptor mice that received MLN cells from PBS_prev_ animals treated with isotype control antibody and then were sensitized; e) Receptor mice that received MLN cells from CMP_prev_ animals treated with isotype control antibody and then were sensitized. Results are representative of two independent experiments with similar results. Two-way ANOVA was used because all the data had normal distribution and equal variances ***P<0.001, **P<0.01, *P<0.05.

Spleen CD4^+^CD25^-^ cells were sorted from naïve mice and stimulated with anti-CD3/anti-CD28, TGF-β and IL-2 during 5 days. Almost 50% of CD4^+^CD25^-^ sorted cells were differentiated into CD4^+^CD25^+^FoxP3^+^ cells (Fig 5A), which secreted 173.68±5.21pg/ml of IL-10 (CD4^+^CD25^-^ spleen cells produced 17.87±1.2pg/ml of IL-10). These cells were injected into receptor naïve mice (1x10^6^ cells) which were subsequently sensitized. After the oral challenge, mice exhibited lower clinical scores, negative skin test and lower serum CMP-specific IgE (Fig 5Bc), compared with control mice that received PBS_iv_ prior to sensitization (Fig 5Bb). An additional control consisted of naïve mice that only received PBS_iv_ and displayed a negative skin test, and low clinical score and serum CMP-specific IgE (Fig 5Ba).

#### *In vivo*-induced regulatory T cells

MLN cells of PBS_prev_ or CMP_prev_ mice were isolated and analyzed for the presence of Tregs. We found a higher percentage of CD4^+^CD25^+^FoxP3^+^ cells in CMP_prev_ compared to PBS_prev_ mice (Fig 5C). Cells were incubated with a-CD25 or isotype control for CD25 depletion (including Treg) (Fig 5D), and then were *iv* administered into naïve recipient mice (4x10^6^ cells), which were subsequently sensitized. As additional control, naïve mice received PBS_iv_ prior to sensitization.

We observed in mice that received PBS and were then sensitized, that the oral challenges induced high clinical scores, positive skin tests and serum specific IgE antibodies (Fig 5Ea). Similarly, mice that were transferred with MLN cells from sensitized animals (PBS_prev_) that were treated with a-CD25 or isotype control (Fig 5Eb and c), or mice that received a-CD25-treated MLN cells from tolerized animals (CMP_prev_) (Fig 5Ed) exhibited hypersensitivity symptoms, positive skin test and high levels of serum specific IgE. Finally, naïve animals that were transferred with isotype-treated cells from tolerized mice (CMP_prev_) showed low clinical score, negative skin tests and serum specific IgE antibodies (Fig 5Ee).

In addition, MLN cells of CMP_prev_ mice stimulated with CMP rendered 587±23 pg/ml of IL-10 and 1558±257pg/ml of TGF-β, while MLN cells from control mice incubated with CMP rendered 121±35 pg/ml of IL-10 and 88±14pg/ml.

## Discussion

Strict allergen avoidance is currently the primary therapy for managing IgE-mediated and non-IgE-mediated food allergy, but in the last two decades there has been increasing interest in optimizing mucosal immunotherapies [8]. Compelling evidences indicate that a step-dose administration of milk proteins through the oral route in patients induces immune tolerance [21]. The immunologic mechanisms underlying OIT is not fully understood. Although there is great heterogeneity in the design of these studies (age, symptoms, randomization, duration, etc.), it has been shown that the cumulative dose of food allergens that patients can tolerate is 200-fold higher for treated patients (2000–10000 mg/day) than placebo-treated patients (40–80 mg/day) [11,22–25]. However, adverse reactions still remain as the main drawback of treatments. For this reason, restitution of tolerance may be the future for food allergy treatment in patients who do not naturally achieve tolerance [26,27], in patients who show an impaired oral and/or systemic tolerance [28], or in patients with difficulties in avoiding the allergenic food. Although oral immunotherapy show promise for IgE-mediated food allergy in clinical trials, it is not ready for implementation in clinical practice. In this sense, animal models could make relevant contributions for medical research. In our study, using an IgE-mediated food allergy mouse model, we provided evidence that the oral administration of low doses of allergens induced CD4^+^CD25^+^FoxP3^+^ cells in the intestinal mucosa, with an increased production of IL-10 and a concomitant suppression of IL-5, IL-13, IgE production and Teff responses. Remarkably, the activation of skin mast cells was abrogated, and the symptoms following an oral exposure to the food allergen were long-term controlled, which may suggest that the induced Tregs controlled mast cell activation or mast cells had low level of bound-IgE. Furthermore, in this work tolerance was achieved with 10 mg of CMP in the preventive protocol, while 10 μg were used in the treatment protocol to reduce the risk of adverse reactions. Most importantly, a higher dose (8-fold increased) of allergen was necessary to promote hypersensitivity symptoms in treated mice compared to sensitized animals. Since animals tolerated an oral challenge with 10 mg of CMP five months after discontinuation of treatment, we can reasonable suggest that acquisition of tolerance was achieved through active suppression. Nevertheless, further studies are essential to understand this long-term management. In this sense, it would be interesting to analyze if the tolerogenic protocols here employed involve the B cell subset. This is a controversial field and it has been demonstrated that the mucosal administration of antigens induces a B cell-dependent T cell-mediated tolerance [29–31].

Studies conducted by Adel-Patient et al. [32] showed that animals preventively treated with oral β-lactoglobulin, and then intraperitoneally sensitized with the same allergen and alum, had a high frequency of CD4^+^CD25^+^FoxP3^+^ cells in MLN, Peyer´s patches and spleen 72 hours after the oral challenge. Yamada et al. [33] described in a rhinitis model that IL-10-expressing CD4^+^CD25^+^FoxP3^+^ T cells were produced in cervical lymph nodes of OVA-sensitized mice that received sublingual immunotherapy with OVA. In other study, Rupa et al. [34] found in orally sensitized mice with egg allergens, that the oral administration of peptide-containing T epitopes of ovomucoid promoted the induction of CD4^+^CD25^+^ and CD4^+^FoxP3^+^ cells in blood, with an augmented secretion of IL-10 and TGF-β in Ag-stimulated spleen cells. However, no markers of tolerance were shown in the intestinal mucosa. In our work, we observed an increased frequency of regulatory T cells in the gut mucosa to dampen the local inflammation. FoxP3 induces the expression of the anti-inflammatory cytokine IL-10 and supports the maintenance of immunosuppressive milieu [35]. A deregulation of FoxP3+ Treg seems to play an important role in allergic disease since a Treg impaired function has been associated with development of allergy [28,36,37], and mutations in the foxp3 gene induce a severe autoimmunity, polyendocrinopathy and allergy (IPEX) [38]. In our study, the adoptive transfer of Treg prevented the further sensitization with CT and CMP, whereas Treg depletion abrogated this immunomodulation. Syed et al described that oral immunotherapy with peanut allergens was associated with an absence of response to the antigen after a month of therapy. They observed a modification of the FoxP3 methylation pattern and Treg activation [39].

Induction of adaptive Treg (iTreg) and increased secretion of IL-10 and TGF-β have been reported in several studies that employed different antibody- or antibody fraction-based biologicals to prevent or control experimental autoimmunity, through not fully understood mechanisms [40–42]. In our study, the controlled mucosal administration of antigens rendered an intestinal tolerogenic environment which induced iTreg that prevented or reversed the T cell-mediated allergic immune response. This approach may provide a therapeutic alternative to induce active suppression for controlling autoimmune and allergic disorders.

In conclusion, we studied two tolerogenic therapeutic strategies that ameliorated the hypersensitivity symptoms following an oral exposure in sensitized mice. We demonstrated that IL-10- and TGF-β-producing CD4^+^CD25^+^FoxP3^+^ mucosal cells were induced with these immunotherapies, thus creating a dominant tolerogenic intestinal environment that suppressed the production of IL-5, IL-13 and IgE.

## Abbreviations

CMP: cow's milk protein
CT: cholera toxin
FoxP3: Forkhead box protein 3
Treg: regulatory T cells
OIT: oral immunotherapy
ig: intragastric
iv: intravenous
EAST: Enzyme allergo sorbent test
